# Where is the human in the data? A guide to ethical data use

**DOI:** 10.1093/gigascience/giy076

**Published:** 2018-06-28

**Authors:** Angela Ballantyne

**Affiliations:** Department of Primary Health Care and General Practice, University of Otago, Wellington, New Zealand

**Keywords:** ethics, big data, data analytics, data protection

## Abstract

Being asked to write about the ethics of big data is a bit like being asked to write about the ethics of life. Big data is now integral to so many aspects of our daily lives—communication, social interaction, medicine, access to government services, shopping, and navigation. Given this diversity, there is no one-size-fits-all framework for how to ethically manage your data. With that in mind, I present seven ethical values for responsible data use.

## Introduction

Data is ubiquitous because it is so useful. This means that many different parties—data subjects and sources, associated communities, researchers, governments, and businesses—will have competing interests in relation to the data. Just as we make trade-offs in our daily life (to walk or to drive to work? doughnut vs. salad for lunch?), we need to make trade-offs about competing interests in relation to data.

I am talking about interests, rather than rights. Note that many parties who don't have legal rights to control access to and use of data, may nonetheless have compelling interests in the data. Responsible data use requires attention to these broad interests. Facebook's recent troubles highlight this. Even if Facebook was legally entitled to share users’ data with Cambridge Analytica, Facebook massively underestimated users’ interests and expectations in relation to privacy, control, and appropriate use.

In areas of rapid progress, such as data science, practice can quickly outstrip the legal framework. Data use may be within the parameters of the law (e.g., data protection or privacy regulation) but may nonetheless be unethical and/or outside the social licence. We should be aiming to align the social licence, ethics, and the law to ensure that data use is publicly acceptable, normatively justified, and legal. Where there is misalignment of the law, ethics, and the social licence, data users need to tread carefully.

## Ethical deliberation

**Figure 1:**
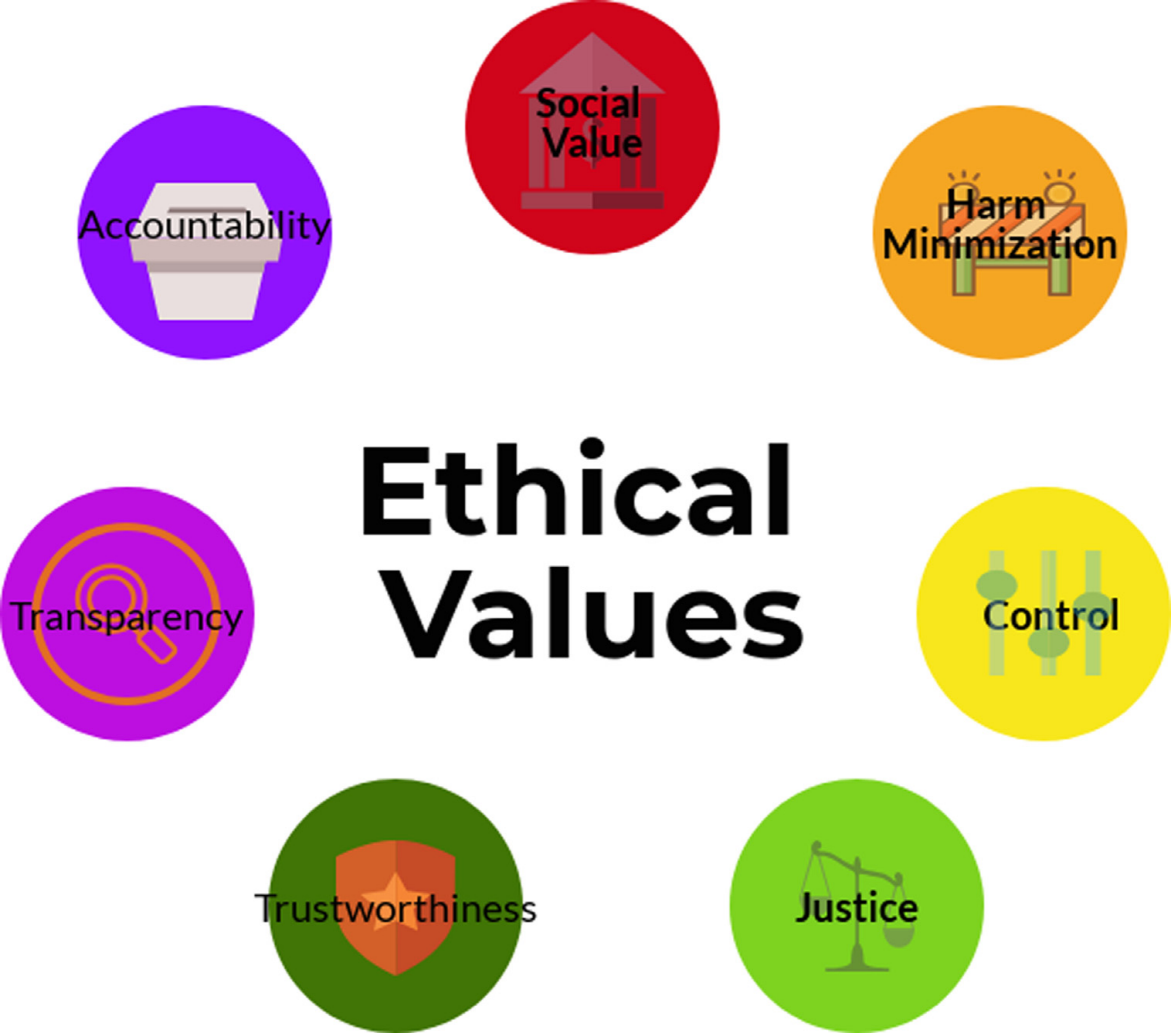
An infographic summarizing the ethical values.

Following is a list of ethical values, also depicted in Fig. 1, that can help identify who has an interest in the data and where these interests might clash; help data holders to articulate the ethical trade-offs that need to be made; and guide deliberation about responsible data use. The values often clash—maximizing data security will conflict with maximizing social value through broader data use. Under different circumstances, priority will appropriately be given to different values. This process is about making informed, explicit, and justifiable trade-offs, rather than following a set of prescribed rules.

### Social value

Data is in demand because it has value. Data can contribute to knowledge and innovation, drive efficiency, reduce harm from ineffective or poorly targeted services, and reduce costs. Open data is important to drive the advancement of scientific knowledge, preserve datasets, test and verify conclusions, refine algorithms, and safeguard against misconduct.

### Harm minimization

Data collection, storage, and use should be designed to minimize and manage risks of harm. Harms can be physical, economic, psychological, or reputational and can be experienced by individuals, communities, or organizations. Anonymization (pseudonymization and de-identification) has been the cornerstone of protecting individual data subjects from harm. However, anonymization is failing in the era of big data, where there are hundreds of thousands of data points for a single individual [1]. Data scientists have proven repeatedly that they can re-identify individuals in supposedly anonymous datasets [2]. Furthermore, anonymization and de-identification do little to protect communities from harm. Data analytics and artificial intelligence (AI) are increasingly used to characterize the behavior of communities and inform the delivery of services. Data can be used to stigmatize or discriminate.

### Control

Control refers to the capacity for data subjects to be autonomous and self-determining. Were data subjects asked for their consent at the point of data collection? To what degree will data subjects’ preferences determine how the data is used? Is this a secondary use of the data that differs from the original consent? Is the data use novel and original or is it likely to be consistent with the expectations of data subjects? Various models of consent have been proposed for data, including broad consent, dynamic consent [3], and meta-consent [4]. However, much data use (especially linking and secondary uses) occurs without consent. In these cases, data users need to be safe stewards of the data. Transparency, engagement, and accountability are especially important for data used without consent [5].

### Justice

Justice concerns the equitable treatment of those with an interest in the data activities, including the fair distribution of any benefits and burdens arising from the collection, storage, use, linkage, and sharing of data. The term “benefit sharing” was first used in relation to non-human genetic resources in the Convention on Biological Diversity adopted at the Earth Summit in Rio de Janeiro, Brazil, in 1992. Benefit sharing requires that the advantages/profits derived from the data are shared fairly among the data providers and the community from which the data originates. Recent data advocacy, especially in relation to indigenous data, has moved away from “benefit sharing” toward “power sharing,” arguing that data subjects and communities should have decision-making capacity in relation to data governance and use [6].

### Trustworthiness

Trustworthiness is the property of being worthy of trust. It can apply to individuals, organizations, and institutions but also relates to data quality, systems of knowledge production, scientific integrity, and professional standards [7]. When judging trustworthiness, we look for truthfulness, reliability, and consistency but also goodwill. A robust data ecosystem requires a high level of trust. A breach of trust can affect not only the agents involved but an entire profession or institution. The dispute between Arizona State University and members of the Havasupai Indian tribe over the use of genetic samples for research left a legacy of mistrust and fear of exploitation [8]. As Tuhiwai Smith famously argued, “’Research’ is probably one of the dirtiest words in the indigenous world's vocabulary” [9]. Trust, when lost, can take significant efforts to rebuild [10].

### Transparency

Transparency is openness and accessibility in decision making and actions. When the data activity occurs without the data subjects’ consent and is justified on the grounds of “social value,” the arguments in favor of transparency and openness are especially compelling. Transparency helps to demonstrate respect for data subjects and trustworthiness, and it underpins public engagement and accountability. Full transparency would include a public description of the data activity, purpose and justification, anticipated social value, harm-mitigation strategies, public engagement strategies, level of security and encryption, research results, and the coding/algorithms. When launching a £1.5 billion initiative in AI in April 2018, France's President Macron announced that anyone receiving AI funding money from the government will be required to make their algorithms open and transparent.

### Accountability

Accountability refers to holding data users and custodians responsible for the consequences of their decisions and actions. Data regulation is increasingly focused on accountability. A significant innovation in the EU General Data Protection Regulation (GDPR) (which came into force in May 2018) is the introduction of “accountability” (Article 5(2)) to the list of principles relating to personal data. Under the GDPR, organizations will need to be more intentional about their data collection and use and maintain open lines of communication with data subjects.

## Conclusion

Given these competing values, there will be multiple different “ethical” solutions to data management. The task is to identify the ethical issues, reason through how to balance conflicting demands, articulate the trade-offs, and justify the conclusions. Do this as publically and transparently as possible, and make time to revise and re-assess.

We use data to tell stories, to make sense of the world. This means telling stories about people and how they live. Data has the appealing veneer of scientific objectivity, but the process of telling stories is never ethically neutral. Our starting point should be to ask: Where is the human in the data? What would this data use look like from the data subjects’ perspective?

## Abbreviations

AI: artificial intelligence; GDPR: General Data Protection Regulation.

## Competing interests

The author declares that she has no competing interests.

## Supplementary Material

GIGA-D-18-00208_Original_Submission.pdf

## References

[1] Roy M Data anonymization techniques less reliable in era of big data. TechTarget. 2017 https://searchcompliance.techtarget.com/feature/High-dimensional-info-complicates-data-anonymization-techniques Accessed 10 May 2018.

[2] Montjoye AJ, Radaelli L, Singh VK, Unique in the shopping mall: on the reidentifiability of credit card metadata. Science. 2015;347:536–9. 25635097 10.1126/science.1256297

[3] Kaye J, Whitley EA, Lund D, Dynamic consent: a patient interface for twenty-first century research networks. Eur J Hum Genet. 2015;23(2):141–6. 24801761 10.1038/ejhg.2014.71 PMC4130658

[4] Ploug T, Holm S Meta Consent - a flexible solution to the problem of secondary use of health data. Bioethics. 2016;30(9):721–32. 27628305 10.1111/bioe.12286 PMC5108479

[5] Ballantyne A, Schaefer GO Consent and the ethical duty to participate in health data research. J Med Ethics. 2018;44 (6):392–396. 29358219 10.1136/medethics-2017-104550

[6] Kukutai T, Taylor J Data sovereignty for indigenous peoples: current practice and future needs. In: Kukutai T Taylor J, ed. Indigenous Data Sovereignty: Toward an Agenda. Acton, Australia: ANU Press; 2016; 1–22. Retrieved from https://press.anu.edu.au/.

[7] Aitken M, Cunningham-Burley S, Pagliari C Moving from trust to trustworthiness: experiences of public engagement in the Scottish Health Informatics Programme. Science & Public Policy. 2016;43(5):713–23. 28066123 10.1093/scipol/scv075 PMC5210028

[8] Mello MM, Wolf LE The Havasupai Indian tribe case–lessons for research involving stored biologic samples. N Engl J Med. 2010;363(3):204–7. 20538622 10.1056/NEJMp1005203

[9] Tuhiwai Smith L Decolonizing Methodologies: Research and Indigenous Peoples. London: University of Otago Press; 1999.

[10] Carter P, Laurie GT, Dixon-Woods M The social licence for research: why care.data ran into trouble. J Med Ethics. 2015;41(5):404–9. 25617016 10.1136/medethics-2014-102374 PMC4431337

