# Supplementary material for: Where is the human in the data? A guide to ethical data use
Source: Gigascience. 2018 Jun 28;7(7):giy076. doi: 10.1093/gigascience/giy076 (PMC6047448; doi:10.1093/gigascience/giy076)

GigaScience

Where is the human in the data? A guide to ethical data use

--Manuscript Draft--

**Manuscript Number:**	GIGA-D-18-00208
**Full Title:**	Where is the human in the data? A guide to ethical data use
**Article Type:**	Commentary
**Funding Information:**	Marsden Fund Fast Start Grant (UOO1515) — Dr Angela Ballantyne
**Abstract:**	Being asked to write about the ethics of big data is a bit like being asked to write about the ethics of life. Big data is now integral to so many aspects of our daily lives – communication, social interaction, medicine, access to government services, shopping and navigation. Given this diversity, there is no one-size-fits-all framework for how to ethically manage your data.
**Corresponding Author:**	Angela Ballantyne<br><br>NEW ZEALAND
**Corresponding Author Secondary Information:**	
**Corresponding Author's Institution:**	
**Corresponding Author's Secondary Institution:**	
**First Author:**	Angela Ballantyne
**First Author Secondary Information:**	
**Order of Authors:**	Angela Ballantyne
**Order of Authors Secondary Information:**	
**Additional Information:**	
**Question**	**Response**
Are you submitting this manuscript to a special series or article collection?	No
**Experimental design and statistics**<br><br>Full details of the experimental design and statistical methods used should be given in the Methods section, as detailed in our Minimum Standards Reporting Checklist. Information essential to interpreting the data presented should be made available in the figure legends.<br><br>Have you included all the information requested in your manuscript?	Yes
**Resources**<br><br>A description of all resources used, including antibodies, cell lines, animals and software tools, with enough	Yes

information to allow them to be uniquely identified, should be included in the Methods section. Authors are strongly encouraged to cite Research Resource Identifiers (RRIDs) for antibodies, model organisms and tools, where possible.<br><br>Have you included the information requested as detailed in our Minimum Standards Reporting Checklist?	
**Availability of data and materials**<br><br>All datasets and code on which the conclusions of the paper rely must be either included in your submission or deposited in publicly available repositories (where available and ethically appropriate), referencing such data using a unique identifier in the references and in the “Availability of Data and Materials” section of your manuscript.<br><br>Have you have met the above requirement as detailed in our Minimum Standards Reporting Checklist?	Yes

Manuscript

Click here to view linked References

Click here to access/download;Manuscript;Editorial GigaScience - Ballantyne 01.06.2018_SCE_NN-AB.docx

FINAL 01 JUNE 2018

Title: "Where is the human in the data? A guide to ethical data use"

Angela Ballantyne[1,*]

1. Department of Primary Health Care and General Practice at University of Otago, Wellington, New Zealand.

*corresponding author
Email: angela.ballantyne@otago.ac.nz

ORCID: 0000-0003-2666-9557

Keywords: Ethics, Big Data, Data Analytics, Data Protection

Abstract:

Being asked to write about the ethics of big data is a bit like being asked to write about the ethics of life. Big data is now integral to so many aspects of our daily lives – communication, social interaction, medicine, access to government services, shopping and navigation. Given this diversity, there is no one-size-fits-all framework for how to ethically manage your data. Wih that in mind, here I attempt to present seven ethical values for responsible data use.

Body Text:

Data is ubiquitous because it is so useful. This means that many different parties – data subjects and sources, associated communities, researchers, governments and businesses – will have competing interests in relation to the data. Just as we make trade-offs in our daily life (to walk or to drive to work? doughnut versus salad for lunch?) we need to make trade-offs about competing interests in relation to data.

I am talking here about interests, rather than rights. Note that many parties who don't have legal rights to control access to and use of data, may none-the-less have compelling interests in the data. Responsible data use requires attention to these broad interests. Facebook's recent troubles highlight this. Even if Facebook was legally entitled to share users' data with Cambridge Analytica, Facebook massively under-estimated users' interests and expectations in relation to privacy, control and appropriate use.

In areas of rapid progress, such as data science, practice can quickly outstrip the legal framework. Data use may be within the parameters of the law (e.g. data protection or privacy regulation), but may nonetheless be unethical and/or outside the social licence.[i] We should be aiming to align the social licence, ethics and the law to ensure that data use is publicly acceptable, normatively justified and legal. Where there is misalignment of the law, ethics, and the social licence, data users need to tread carefully.

The following is a list of ethical values, also depicted in Figure 1, that can: (1) help identify who has an interest in the data and where these interests might clash; (2) help data holders to articulate the

---

[i] 'Ethics' is normative – is makes a claim about what the morally correct course of action would be, and attributes praise or blame. 'Social licence' is descriptive – it describes whether a given data use is accepted by the data subjects, public, and other stakeholders.

ethical trade-offs that need to be made; and (3) guide deliberation about responsible data use. The values often clash – maximising data security will conflict with maximising social value through broader data use. In different circumstances priority will appropriately be given to different values. This process is about making informed, explicit and justifiable trade-offs, rather than following a set of prescribed rules.

Social value Data is in demand because it has value. Data can contribute to knowledge and innovation, drive efficiency, reduce harm from ineffective or poorly targeted services and reduce costs. Open data is important to drive the advancement of scientific knowledge, preserve datasets, test and verify conclusions, refine algorithms, and safeguard against misconduct.

Harm minimization Data collection, storage and use should be designed to minimise and manage risks of harm. Harms can be physical, economic, psychological or reputational and can be experienced by individuals, communities or organisations. Anonymization (pseudonymization and de-identification) has been the cornerstone of protecting individual data subjects from harm. But anonymization is failing in the era of big data, where there are hundreds of thousands of data points for a single individual.[1] Data scientists have proved repeatedly that they can re-identify individuals in supposedly anonymous data sets.[2] Furthermore, anonymization and de-identification do little to protect communities from harm. Data analytics and AI are increasingly used to characterize the behaviour of communities and inform the delivery of services. Data can be used to stigmatize or discriminate.

Control Control refers to the capacity for data-subjects to be autonomous and self-determining. Were data subjects asked for their consent at the point of data collection? To what degree will data subjects' preferences determine how the data is used? Is this a secondary use of the data that differs from the original consent? Is the data use novel and original or is it likely to be consistent with the expectations' of data subjects? Various models of consent have been proposed for data – including broad consent, dynamic consent[3] and meta-consent[4]. However much data use (especially linking and secondary uses) occurs without consent. In these cases, data users need to be safe stewards of the data. Transparency, engagement and accountability are especially important for data used without consent. [5]

Justice Justice concerns the equitable treatment of those with an interest in the data activities; including the fair distribution of any benefits and burdens arising from the collection, storage, use, linkage and sharing of data. The term 'benefit sharing' was first used in relation to non-human genetic resources in the Convention on Biological Diversity (CBD) adopted at the Earth Summit in Rio de Janeiro, Brazil in 1992. Benefit sharing requires that the advantages/profits derived from the data are shared fairly amongst the data providers and to the community from which the data originate. Recent data advocacy, especially in relation to indigenous data, has moved away from 'benefit sharing' towards 'power sharing' – arguing that data subjects and communities should have decision making capacity in relation to data governance and use.[6]

Trustworthiness Trustworthiness is the property of being worthy of trust - it can apply to individuals, organisations, and institutions, but also relates to data quality, systems of knowledge production, scientific integrity and professional standards.[7] When judging trustworthiness, we look for truthfulness, reliability, and consistency but also goodwill. A robust data ecosystem requires a high-level of trust. A breach of trust can affect not only the agents involved, but an entire profession or

institution. The dispute between Arizona State University and members of the Havasupai Indian tribe, over the use of genetic samples for research left a legacy of mistrust and fear of exploitation.[8] As Smith famously argued "'Research' is probably one of the dirtiest words in the indigenous world's vocabulary."[9] And trust, when lost, can take significant efforts to rebuild.[10]

Transparency Transparency is openness and accessibility in decision-making and actions. When the data activity occurs without the data subjects' consent and is justified on the grounds of 'social value', the arguments in favour of transparency and openness are especially compelling. Transparency helps to demonstrate respect for data subjects and trustworthiness; and it underpins public engagement and accountability. Full transparency would include a public description of the data activity, purpose and justification, anticipated social value, harm-mitigation strategies, public engagement strategies, level of security and encryption, research results, and the coding/algorithms. When launching a £1.5 Billion initiative in AI in April 2018, President Macron announced that anyone receiving AI funding money from the French government will be required to make their algorithms open and transparent.

Accountability Accountability refers to holding data users and custodians responsible for the consequences of their decisions and actions. Data regulation is increasingly focused on accountability. A significant innovation in the EU General Data Protection Regulation (GDPR) (which will come into force on the 25th May 2018) is the introduction of 'accountability' (Article 5(2)) to the list of principles relating to personal data. Under the GDPR organisations will need to be more intentional about their data collection and use and maintain open lines of communication with data subjects.

Given these competing values, there will be multiple different 'ethical' solutions to data management. The task is to identify the ethical issues, reason though how to balance conflicting demands, articulate the trade-offs and justify the conclusions. Do this as publically and transparently as possible; and make time to revise and re-assess.

We use data to tell stories, to make sense of the world. This means telling stories about *people* and how they live. Data has the appealing veneer of scientific objectivity; but the process of telling stories is never ethically neutral. Our starting point should be to ask: Where is the human in the data? What would this data use look like from the data subjects' perspective?

Abbreviations

AI: Artificial Intelligence; CBD: Convention on Biological Diversity; GDPR: General Data Protection Regulation

Declarations

Competing interests The author declares that they have no competing interests.

Funding Marsden Fund Fast Start Grant (UOO1515) 2016-2019 "The ethics of research on clinical data and tissue without explicit patient consent"

Acknowledgements

I would like to thank the *Working Group for Big Data Ethics in Health and Research* who are a delight to work with and who contributed to the definitions of the values used here. The Working Group is organized by the Science, Health and Policy-Relevant Ethics (SHAPES) initiative at the Centre for Biomedical Ethics, Yong Loo Lin School of Medicine at the National University of Singapore. Co-Chairs: Prof Tai E Shyong (National University of Singapore/National University Hospital) and Prof Graeme Laurie (University of Edinburgh). Members: Dr Angela Ballantyne (University of Otago); Dr Iain Brassington (University of Manchester); Mr Markus Labude (National University of Singapore); A/Prof Hannah Lim Yee Fen (Nanyang Technological University); A/Prof Wendy Lipworth (University of Sydney); Dr Tamra Lysaght (National University of Singapore); Dr Owen Schaefer (National University of Singapore); Prof Cameron Stewart (University of Sydney); A/Prof Shirley Sun Hsiao-Li (Nanyang Technological University); and Dr Vicki Xafis (National University of Singapore).

Thanks also to those who attended the Data Ethics Governance Workshop in Wellington NZ in 2017, for fruitful discussion of these topics, especially Maria Stubbe, June Atkinson and Rochelle Style.

Figure legend

Figure 1: An infographic summarizing the ethical values

References.

[1] Roy M. Data anonymization techniques less reliable in era of big data. TechTarget. 2017. https://searchcompliance.techtarget.com/feature/High-dimensional-info-complicates-data-anonymization-techniques Accessed 10 May 2018.
[2] Montjoye AJ, Radaelli L, Singh VK, Pentland A. Unique in the shopping mall: On the reidentifiability of credit card metadata. Science. 2015; 536-539.
[3] Kaye J, Whitley EA, Lund D, Morrison M, Teare H, Melham K. 2015. Dynamic consent: a patient interface for twenty-first century research networks. Eur J Hum Genet. 2015 Feb;23(2):141-6. doi: 10.1038/ejhg.2014.71
[4] Ploug T, Holm S.. Meta Consent - A Flexible Solution to the Problem of Secondary Use of Health Data. Bioethics. 2016; 30(9):721-732. doi: 10.1111/bioe.12286.
[5] Ballantyne A, Schaefer GO. Consent and the ethical duty to participate in health data research. J Med Ethics. 2018. doi: 10.1136/medethics-2017-104550.
[6] Kukutai T, Taylor J. Data sovereignty for indigenous peoples: current practice and future needs. In T. Kukutai, & J. Taylor (Eds.), Indigenous Data Sovereignty: toward an agenda (pp. 1-22). 2016. Acton, Australia: ANU press. Retrieved from https://press.anu.edu.au/
[7] Aitken M, Cunningham-Burley S, Pagliari C. Moving from trust to trustworthiness: Experiences of public engagement in the Scottish Health Informatics Programme. Science & Public Policy. 2016; 43(5):713-723. doi:10.1093/scipol/scv075.
[8] Mello MM, Wolf LE. The Havasupai Indian tribe case--lessons for research involving stored biologic samples. N Engl J Med. 2010 Jul 15; 363(3):204-7. doi: 10.1056/NEJMp1005203.
[9] Tuhiwai Smith, Linda, 1999. Decolonizing Methodologies : Research and Indigenous Peoples. London: University of Otago Press.
[10] Carter P, Laurie GT, Dixon-Woods M. The social licence for research: why care.data ran into trouble. J Med Ethics. 2015; 41(5):404-9.

Figure 1

Click here to access/download;Figure;Fig1 Ballantyne.png

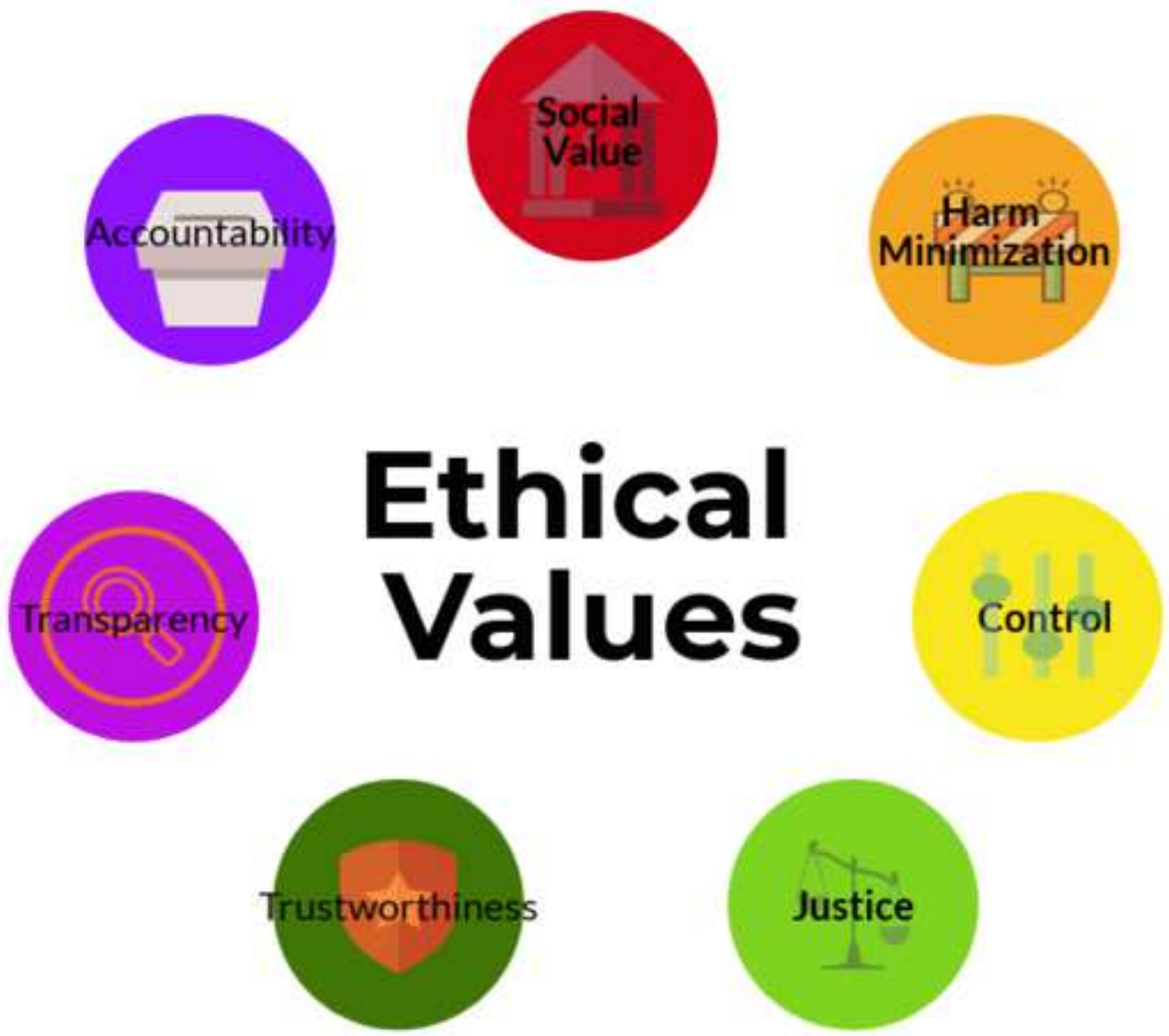

Supplement: GIGA-D-18-00208_Original_Submission.pdf [file giy076_giga-d-18-00208_original_submission.pdf]
